# Bioabsorbable osteofixation for orthognathic surgery

**DOI:** 10.1186/s40902-015-0003-7

**Published:** 2015-02-19

**Authors:** Young-Wook Park

**Affiliations:** Department of Oral and Maxillofacial Surgery, College of Dentistry, Gangneung-Wonju National University, 7 Jukheon-gil, Gangneung, 210-702 Korea

**Keywords:** Orthognathic surgery, Bioabsorbable plate, Biodegradation, Skeletal stability

## Abstract

Orthognathic surgery requires stable fixation for uneventful healing of osteotomized bony segments and optimal remodeling. Titanium plates and screws have been accepted as the gold standard for rigid fixation in orthognathic surgery. Although titanium osteofixation is the most widely used approach, the use of bioabsorbable devices has been increasing recently. Biodegradation of bioabsorbable devices eliminates the need for a second operation to remove metal plates and screws. However, long-term stability and relapse frequency in bioabsorbable osteofixation are still insufficiently studied, especially in cases of segmental movements of great magnitude or segmental movements to a position where bony resistance exists. This paper reviews the background, techniques, and complications of bioabsorbable osteofixation and compares bioabsorbable and titanium osteofixation in orthognathic surgery in terms of skeletal stability.

## Introduction

Successful orthognathic surgery requires a comprehensive surgical plan, effective osteofixation system for long-term skeletal stability, and achieving satisfactory esthetic facial appearance. Recently, bimaxillary orthognathic surgery has become popular because mandibular setback does not completely remove the midfacial symptoms of mandibular prognathism, which is often found in oriental races. Consequently, oral and maxillofacial surgeons need to perform more complicated surgery, which requires major segmental movements, i.e., those of greater magnitude or movements to a position where bony resistance exists.

Recent trends in orthognathic surgery include control of the occlusal plane to reduce the length of the face and application of so called “functional orthognathic surgery”, which means surgery first, orthodontic treatment later for patient’s convenience. These trends result in major bone movements with unstable occlusal interdigitation. Therefore, the need for rigid fixation becomes more important in modern orthognathic surgery. For a long time, titanium plates and screws have been considered to be the “gold standard” for rigid fixation in orthognathic surgery. Although titanium binds to the bone and asymptomatic bone plates can be retained [1], titanium plates should be removed due to growth disturbance, possible hypersensitivity to cold exposure, interference with radiological evaluation, possible stress-shielding effect as well as patients’ requests [2-5].

Oral and maxillofacial surgeons are increasingly using bioabsorbable devices because they eliminate the need for troublesome second operations to remove metal devices. Numerous clinical studies have documented comparable results between the use of resorbable and titanium plates and screws in orthognathic surgery regarding postoperative stability and relapse frequency [6-10]. However, there are few reports concerning postoperative skeletal stability after bioabsorbable osteofixation in a series of orthognathic cases accompanying major maxillomandibular segmental movements [10]. This review highlights the evolution of resorbable osteosynthesis technology and postoperative stability of bioabsorbable osteosynthesis in orthognathic surgery.

## Conformation

### Conformation

The idea of biodegradable plates may have emerged from absorbable sutures. In 1966, polylactic acid, which is a major component of biodegradable polymers, was first proposed for surgical implants [11]. The use of biodegradable materials to stabilize the fractured facial skeleton was first reported in 1971[12]. Since then, resorbable polymeric plates and screws have been used widely in pediatric patients with maxillofacial traumas because permanent fixation might hinder their facial growth [13]. At first, the strength of resorbable plates and screws was poor, but the strength of the devices was increased by using self-reinforcement technology. Finally, encouraging results were reported in the treatment of mandibular fractures [14,15] and orthognathic surgery[16,17]. Recently, the concept has been changed from simply “resorbable” to “bioabsorbable”, which means biodegradation plus stimulation of osteoconduction. Commercially available resorbable and bioabsorbable devices are listed in Table 1.

**Table 1:** **Commercially available resorbable or bioabsorbable devices for osteofixation**

**Product**	**Manufacturer**	**Year of invention**	**Conformation**	**Biodegradation period**
Biofix®	BionX	1984	SR-PGA	6 weeks
Orthosorb®	Depuy	1991	PDS	6 months
FixsorbMX®	Takiron	1994	PLLA	2 years
Lactosorb®	Walter Lorenz	1996	PLLA/PGA	12-18 months
MacroSorb®	Macropore	1999	P-L/D-LA	2 years
ResorbX®	KLS martin	2001	P-L/D-LA	2 years
Inion CPS®	Inion	2001	P-L/D-LA	2 years
BiosorbFX®	Bionix Implants	2001	P-L/D-LA	2 years
PolyMax®	Synthes	2003	P-L/D-LA	2 years
Delta System®	Stryker	2004	P-L/D-LA /GA	2 years
OsteotransMX®	Takiron	2007	u-HA/PLLA	5.5 years
Inion CPS®	Inion	2007	P-L/D-LA/TMC	2 years

SR-PGA: Self-reinforced polyglycolic acid.

PDS: Polydioxanone.

PLLA: Poly-L-lactic acid.

PDLA: Poly-D-lactic acid.

u-HA: unsintered hydroxyapatite.

TMC: trimethylenecarbonate.

**Figure 1:**
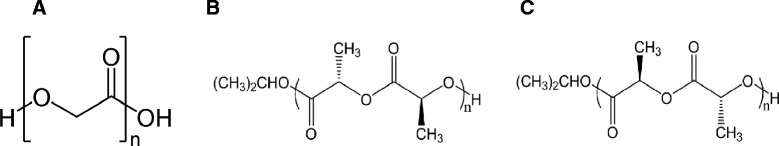
**Structural formulas of polyglycolic acid** **(A)** **, poly-L-lactic acid** **(B)** **, and poly-D-lactic acid** **(C).**

The ideal biodegradable material should not only support bone fragments during healing, but also resorb fully once the healing process is completed, and the resulting metabolites should not cause any local or systemic problems. In addition, the required amounts of such material must be small and it must be flexible to be applied at variable maxillofacial bone sites. Three resorbable materials, polyglycolic acid (PGA), poly-l-lactic acid (PLLA), and poly-d-lactic acid (PDLA), have been introduced (Figure 1). Self-reinforced PGA devices are rapidly degradable, semirigid and disappear by 6 weeks after implantation; they are used in neurosurgery due to their low strength [18]. The total resorption time of PLLA is over 3.5 years [19]. A PLLA device, FixsorbMX**®** (Takiron, Osaka, Japan), has been used in facial bone surgery. The reported problems were insufficient intensity of materials, foreign-body reactions and late degradation tissue response [20].

Polymers and copolymers of PGA, PLLA, and PDLA were given preference over pure PGA and PLLA [21]. Lactosorb**®** (Walter Lorenz Surgical Inc., Jacksonville, Florida, USA) is a copolymer of PLLA (82%) and PGA (18%). The copolymer is structured to provide adequate strength for 6–8 weeks and to allow a resorption time of 12–18 months [22]. It is metabolized via the citric acid cycle and is eventually excreted by the lungs as carbon dioxide and water [23].

**Figure 2:**
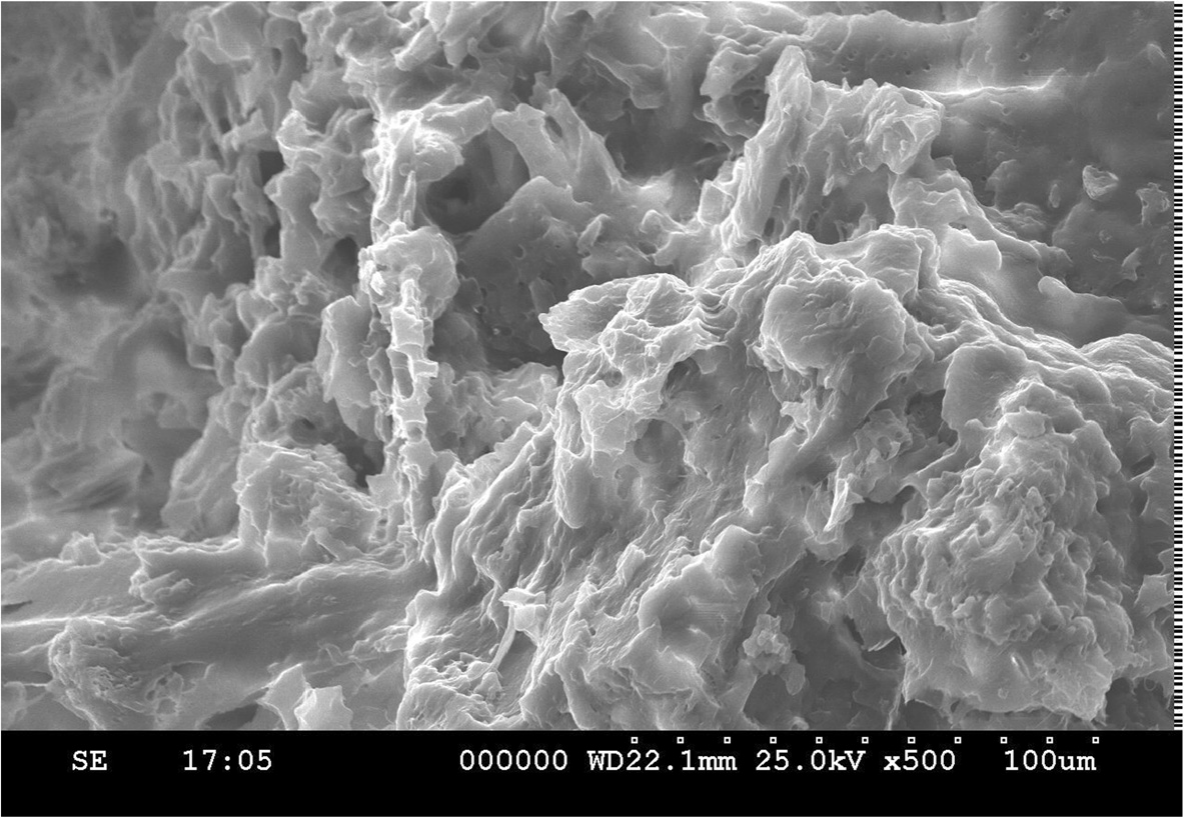
**SEM image showing degrading amorphous 70:30 poly-L/D-lactide copolymer plate, which was explanted 1 year after implantation for osteofixation of mandibular SSRO site.**

BiosorbFX® (Bionix Implants Inc., Tampere, Finland) is a copolymer of d-lactide (30%) and l-lactide (70%). Clinical papers have reported stable results in orthognathic surgery due to its adequate strength [24,25]. Amorphous 70:30 poly-l/d-lactide copolymer plates are hydrolyzed through water penetration into the plate, which breaks the copolymer chains into smaller particles (Figure 2). In contrast to macrocrystalline PLLA structures, hydrolyzed amorphous copolymer can be readily degraded by phagocytic cells to carbon dioxide and water [26].

**Figure 3:**
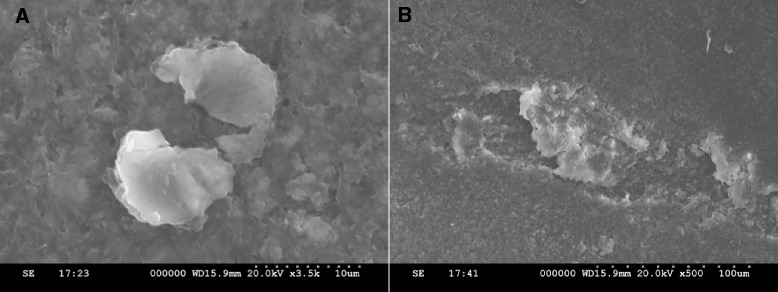
**SEM image showing that a macrophage is attached the surface of u-HA/PLLA nano-composite plate, which explanted 7 months after operation (A). (B)** A defect on the surface of the bioabsorbable plate by macrophage indicates that a process of biodegradation is in progress.

Recently, hydroxyapatite has been incorporated into PLLA because of the documented osteoconductive capacity of the former. OsteotransMX**®** (Takiron) plates are made of a composite material consisting of fine particles of unsintered hydroxyapatite (u-HA) and carbonate ion combined with PLLA. As they are osteoconductive and biodegradable, the u-HA/PLLA nano-composites demonstrate the potential for complete replacement by bony tissue [27]. Furthermore, these devices maintain a bending strength equal to that of human cortical bone for 25 weeks *in vivo* [28]. Once it has been implanted, PLLA starts to be hydrolyzed by body fluids and to undergo biodegradation (Figure 3). The molecular weight of PLLA decreases and the u-HA fraction increases for about 2 years. The PLLA matrix is completely absent from the composites after 4 years, and almost all u-HA particles are replaced with bone after 5.5 years [29]. In comparison with early resorbable polymers, u-HA/PLLA osteoconductive composites provide more stable segment retention in orthognathic surgery [30].

Ballon et al. [31]have conducted a clinical study using a new polymer composition for resorbable osteosynthesis, poly-l/d-lactide-trimethylenecarbonate (TMC) (Inion, Tampere, Finland). P-l/d-lactide-TMC osteosynthesis seemed to have less strength against compressive forces after maxillary elongation and it is less resistant to the forces exerted by the tongue pressing against the mandible after setback [31].

### Clinical Applications

**Figure 4:**
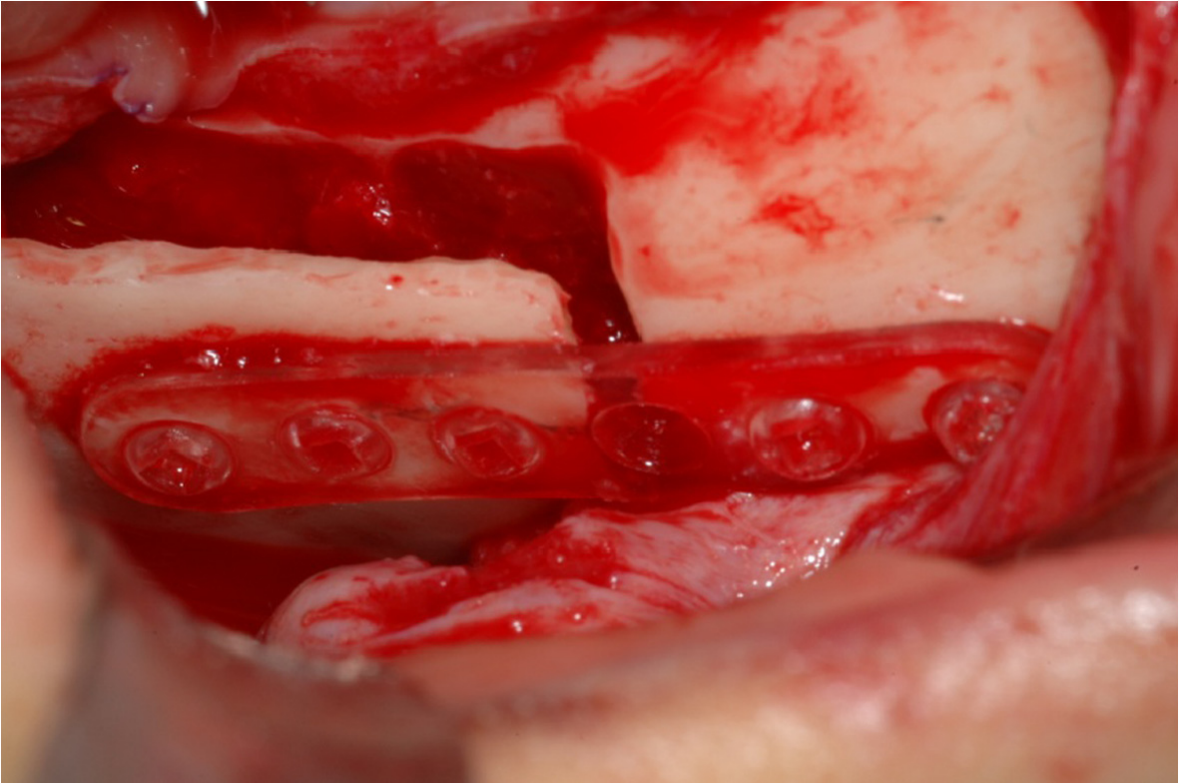
**A 2.4 mm 6-hole poly-L/D-lactide plate is applied for fixation of mandibular SSRO.**

One 2.4-mm, 6-hole poly-l/dl-lactide plate and monocortical screws are applied at the mandibular sagittal split ramus osteotomy (SSRO) site. Three 6-mm screws are engaged into the proximal segments, and two screws are engaged in the distal segment, usually located below the inferior alveolar canal. One hole, located in the osteotomy gap, can be left empty if other screws are sufficiently tight (Figure 4). Other standard methods for osteosynthesis of mandibular SSRO are the triangular placement of bicortical screws [32,33] or the use of two mini-plates and monocortical screws. When two plates are applied, one is located above the inferior alveolar canal, and the other one is below the canal.

**Figure 5:**
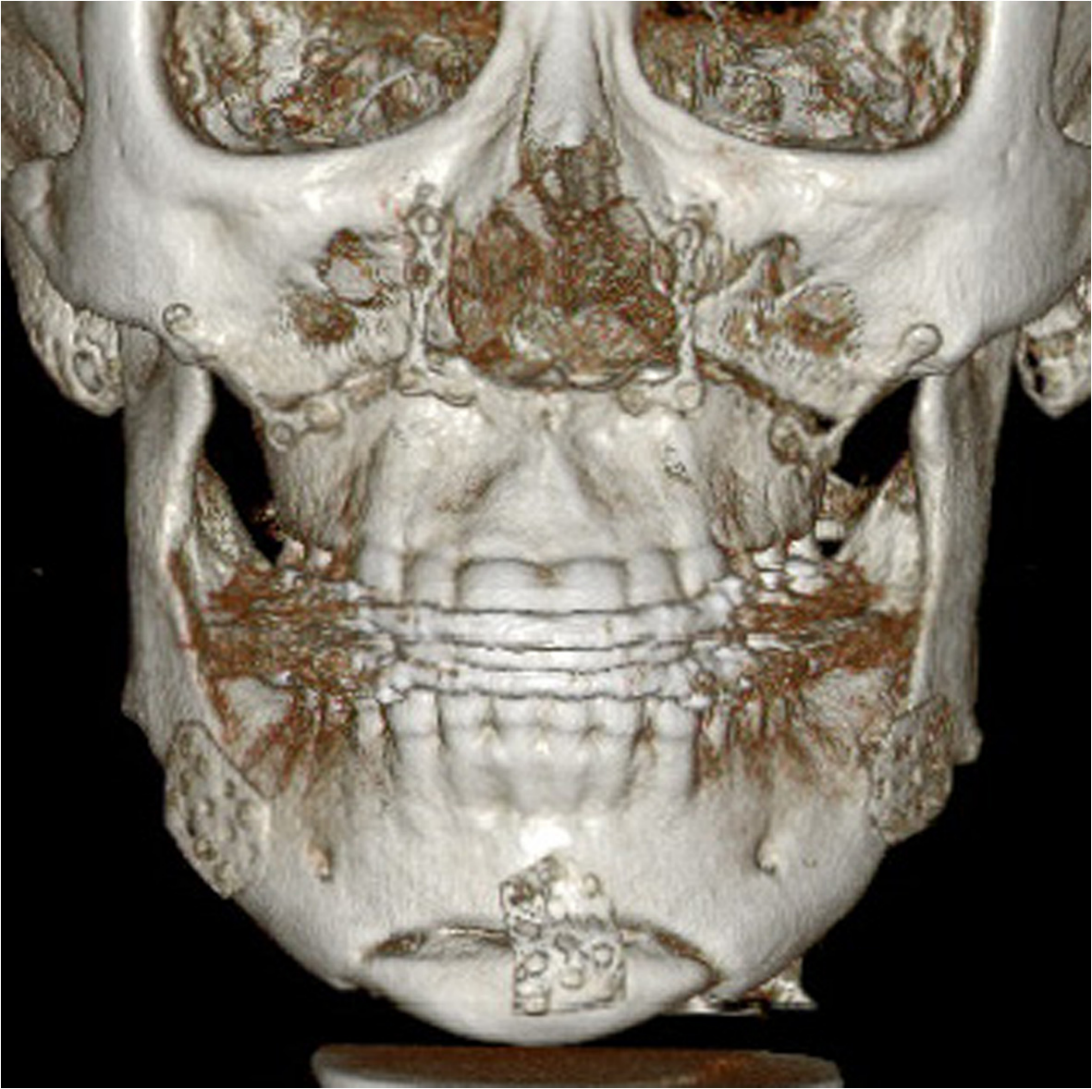
**3-dimensional reconstructed CT image showing maxillomandibular osteotomy with simultaneous genioplasty and fixation with u-HA/PLLA composite bioabsorbable devices in a female patient with mandibular prognathism.** Maxillary osteosynthesis was performed with four L-type mini-sized plates, and mandibular osteosynthesis was performed using bioabsorbable mesh and monocortical screws.

Regardless of the fixation method, mandibular setback remains a more unstable movement than mandibular advancement [34-36]. One mini-resorbable plate for fixation of mandibular SSRO with mandibular setback seems to lead to segment mobility in early postoperative period. A 0.7 mm-thick u-HA/PLLA mesh (Osteotrans-MX**®**) can be also applied after mandibular SSRO, especially when major segmental movements have been performed. Osteosynthesis by using u-HA/PLLA devices in orthognathic surgery is reliable because of their rigidity as well as osteoconductivity and bone-bonding capacity [37]. Mandibular symphyseal osteotomies have also been safely fixed with appropriate bioabsorbable plates and monocortical screws (Figure 5).

The Le Fort I osteotomies are stabilized with four L-shaped resorbable or bioabsorbable plates and secured bilaterally in the pyriform aperture and zygomatic buttress. Reliable results have also been reported with the use of a biodegradable mesh [38,39]. Segmental Le Fort I osteotomy is also stabilized with the above standard fixation, i.e., the use of four 1.2 mm-thick, 7-hole L-type poly-l/d-lactide plates [40].

### Complications

The drawbacks of using resorbable devices include their higher cost and some technical problems due to the characteristics of the material itself, postoperative plate fractures, and development of delayed foreign-body reactions. In 2003, Landes et al. reported that 27% of patients had complications after resorbable plate osteosynthesis of sagittal split osteotomies with major bone movement [41]. The complication rate is now decreasing thanks to the development of new material compositions as well as improvement of surgical skills. Cheung *et al*. have concluded that the introduction of resorbable devices did not lead to an increase in intraoperative morbidities and postoperative complications [42].

Material-specific complications during operation include screw fractures, the need for wider dissection due to the larger sizes of resorbable devices, and difficulties in molding the devices into the desired shape. Screw fractures occur most often in screw heads when excessive force from the screwdriver is applied. A new hole is drilled through the fractured screw or an emergency screw can be used if adequate fixation cannot be obtained with a regular one. The need to increase the dimension for application during surgery has been recently met by using smaller products. Poly-l/d lactide plates and u-HA/PLLA composite plates are easily bendable with fingers at room temperature, combining wave-forms with angles and torsions, and can be maintained in the desired position without heating so far as slower bending and less force are applied. OsteotransMX**®** plates are bendable to 40 degrees at room temperature [30]. Preshaped bent plates are also commercially available, or a boiling-water bath can be used.

**Figure 6:**
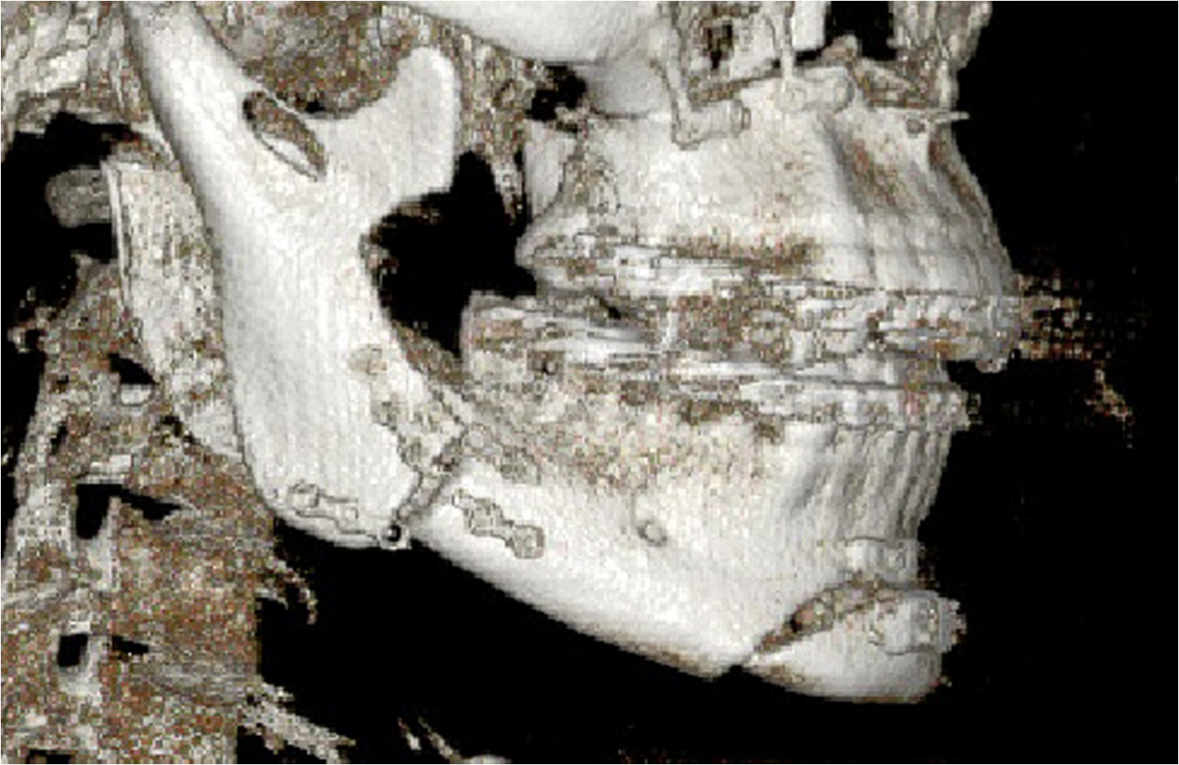
**3-dimensional reconstructed CT image showing that two mini-sized, bioabsorbable plates are fractured 7 weeks after implatation.**

Titanium plates may also be fractured by excessive forces. Fractures of resorbable devices are more problematic when major segmental movements are performed in orthognathic surgery. There are few published clinical studies on the incidence of postoperative plate fractures when resorbable or bioabsorbable plates and screws are used. According to the author’s clinical experience, one 2.4-mm poly-l/d-lactide plate seems to be more stable than two mini-bioabsorbable plates. Only one case of plate fracture was recognized with the use of these plates in a series of 63 bimaxillary orthognathic surgery patients (126 osteofixations) in comparison with 3 cases out of 139 osteofixations when two mini-plates at a SSRO site were used (Figure 6). In a randomized controlled study, the incidence of plate removal was higher in maxillofacial surgery with the biodegradable system in comparison with titanium fixation [43].

**Figure 7:**
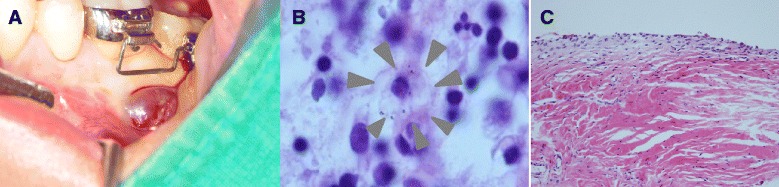
**A case of inflammatory complication.** **(A)** A localized inflammatory lesion was developed 8 months after operation. **(B)** A macrophage contained microorganisms in their cytoplasm (gray arrowheads, H&E, X200). **(C)** Section from the tissue in the vicinity of the u-HA/PLLA plate, showed fibrous scar tissue without inflammatory reaction. In the figure, upper border was in contact with the plate (H&E, X40).

**Figure 8:**
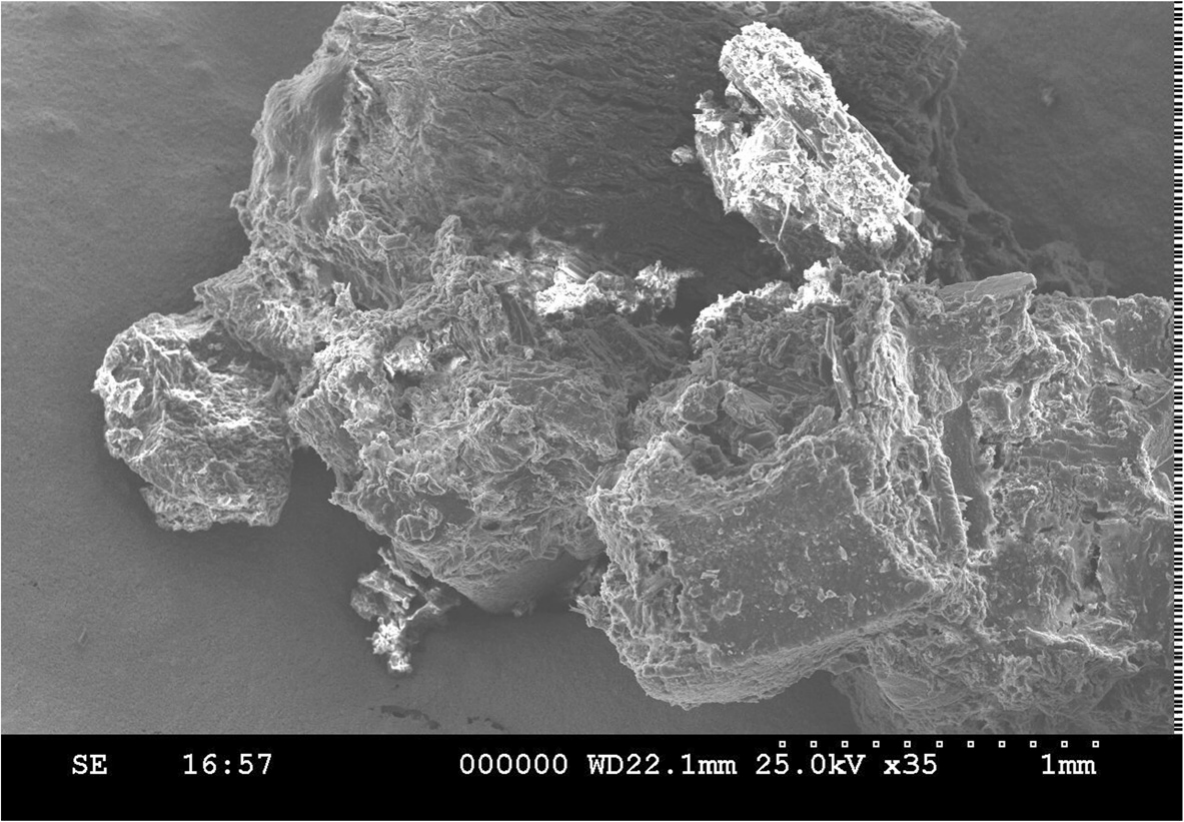
**SEM image showing crystal-like PLLA material internalized by various cells.**

Inflammatory complications may also develop, especially in the mandible. Localized soft tissue inflammation may be associated with a fractured absorbable plate. Intraoral pathogens also induce localized inflammatory lesions around the absorbable devices when inadvertently inoculated. In this case, histological examination demonstrates a granulomatous lesion heavily infiltrated with macrophages and small round cells. However, sections of the tissue in the vicinity of the absorbable plate showed fibrous scar tissue into which few inflammatory cells had infiltrated (Figure 7). Some clinical studies have reported that foreign-body inflammatory reactions develop with the use of PLLA [44,45]. At a later degradation stage, hydrolyzed, disintegrated PLLA undergoes enzymolysis. Remaining crystal-like PLLA particles may trigger the foreign-body inflammatory reaction, although they are not very irritating to the host cells (Figure 8). In addition, an uneven release of PLLA fragments can cause physical inflammatory reaction, but the incidence is rare [29]. These inflammatory lesions can be successfully treated with routine drainage and antibiotics.

### Skeletal Stability

An increasing number of clinical reports concerned with skeletal stability and relapses after bioabsorbable polymer osteosyntheses in orthognathic surgery are found in PubMed. Haers *et al*. reported predictable short-term skeletal stability with the use of poly-l/dl-lactide plates and screws in 10 consecutive cases of bimaxillary procedures with simultaneous genioplasties [46]. Shand *et al*. reported mild mobility of the maxilla in the early postoperative period with the use of Lactosorb**®** bicortical screws for stabilization of mandibular SSRO in bimaxillary orthognathic surgery. However, stability was within normal limits at 6 weeks postoperatively [47].

Mandibular mobility in the early postoperative period was noted, especially when strong elastics were applied immediately postoperatively, but this problem could be easily overcome by gradual application of rubber force and avoidance of occlusal stress. In the author’s experience, none of bimaxillary orthognathic surgery patients (*n* = 153) treated after 2002 had fragment displacement that required refixation. All minor occlusal discrepancies were successfully controlled with elastic bands and guided occlusion. No malocclusions were noted during the follow-up period of at least 2 years. However, Ahn *et al*. reported a higher incidence of complications in patients with resorbable fixation compared with nonresorbable fixation in terms of postoperative anterior open bite and relapse frequency [48].

According to a preliminary report from another group, postoperative osseous movement was small when poly- l/dl-polylactide plates were used [49]. As a result, resorbable osteofixation permitted clinically faster occlusal and condylar setting than titanium osteosynthesis, because segments showed low mobility in cleft lip and palate orthognathic surgery [49]. Other researchers have demonstrated that resorbable devices did not increase segmental mobility or long-term instability compared with titanium plates in orthognathic surgery, including sagittal splitting of mandibular ramus and mandibular advancement [50,51]. In mandibular setback surgery, Paeng *et al*. suggested that bicortical resorbable screws offered a clinically stable outcome except for vertical measurements, compared with titanium fixation (*n* = 25) [52]. Ueki *et al*. concluded that the change in condylar angle after SSRO was greater in the group with titanium fixation than in the group with PLLA fixation (*n* = 20 per group) [53].

In bimaxillary orthognathic surgery, PLLA/PGA plates appear to provide stable osteosynthesis for maxillary advancement of up to 5 mm [54]. In another study on the bimaxillary procedure, a slight tendency for vertical relapse was reported in the group with PLLA osteofixation compared to the titanium osteofixation group, but the differences were not clinically significant and finally normal occlusion was established in both groups [30]. Another retrospective study aimed to determine the differences in postoperative stability between poly-l/d-lactide and titanium plate systems used for fixation in bimaxillary orthognathic surgery, in particular maxillary posterior impaction surgery (*n* = 30) [55]. Six months after surgery, there was no significant difference between the groups, as analyzed by lateral cephalogram. Furthermore, when segmental Le Fort I osteotomy for major movement of maxilla (*n* = 15) in bimaxillary orthognathic surgery was performed, the maxillary position remained stable with resorbable osteosyntheis [40].

No significant difference in the postsurgical relapse rate after mandibular setback surgery was found in the resorbable plate group [56]. Absolute postoperative skeletal instability was not significantly different between resorbable and titanium plating systems for osteofixation in bimaxillary orthognathic surgery [57-59]. In contrast, Landes *et al*. reported compromised segmental stability in maxillary elongation and in mandibular setback with u-HA/PLLA composite osteosynthesis. They applied one 1.4-mm 4-hole plate and 8-mm monocortical screws at a mandibular SSRO site and recommended longer intermaxillary fixation, double osteosynthesis or use of larger plates [30]. However, in most studies the groups were not matched for the magnitude or direction of fragment movement. Prospective studies with larger patient numbers are needed.

## Conclusions

The use of bioabsorbable devices has resolved several problems of titanium fixation, such as the need for a second operation and interference with radiological evaluation. From the literature review, the author concluded that bioabsorbable osteosynthesis systems are reliable for fragment fixation in orthognathic procedures with major maxillomandibular segmental movements. The use of bioabsorbable devices leads to predictable postoperative long-term skeletal stability, which appears to be similar to that provided by titanium devices. In the future, we need less expensive bioabsorbable devices that degrade rapidly in the body.
